# Causal relationship between IgG N-glycosylation and autoimmune thyroid diseases, and the possible mediating role of immune cells and inflammatory cytokines

**DOI:** 10.1097/MD.0000000000045649

**Published:** 2025-10-31

**Authors:** Wenhua Li, Dongni Fan, Dongmi Wei, Wei Wei, Ying Zhang, Min Yi

**Affiliations:** a Department of Pathology, Liuzhou Worker’s Hospital, Liuzhou, China; b Department of Pathology, Liuzhou People’s Hospital, Liuzhou, China.

**Keywords:** autoimmune thyroid diseases, Graves’ disease, IgG N-glycosylation, immune cells, inflammatory cytokines, Mendelian randomization

## Abstract

Immunoglobulin G N-glycans have been associated with the risk of autoimmune thyroid diseases (AITD). In the present study, we investigated the potential causal relationship between IgG N-glycosylation and AITD risk. Employing 2-sample Mendelian randomization (MR) and mediation analysis, we evaluated the causal associations between IgG N-glycosylation and 4 types of AITD, Graves’ disease, autoimmune thyroiditis, autoimmune hyperthyroidism and autoimmune hypothyroidism – using genome-wide association study summary data. Fifteen IgG N-glycan traits were found to have causal relationships with AITD. Moreover, upon considering inflammatory cytokines and immune cell phenotypes as outcomes, 6 inflammatory cytokines and 14 immune cell phenotypes exhibited significant causal relationships with IgG N-glycan traits. Subsequent mediation analyses using 2-step MR revealed that “CD25 on CD24+ CD27+ B cells” mediated the causal association between IGP11 and GD, “HLA DR+ T cell%lymphocyte” mediated the causal association between IGP59 and autoimmune thyroiditis, and “B_NGF” mediated the causal association between IGP59 and autoimmune hyperthyroidism. However, further validation through using multivariable Mendelian randomization (MVMR) indicated that only B_NGF played a mediating role in the causal relationship between IGP59 and autoimmune hyperthyroidism, as other 2 mediators did not yield significant results. This MR study comprehensively assessed the interrelationships among IgG glycosylation, inflammatory cytokines, immune cells, and AITD, identifying potential biomarkers for predicting AITD prognosis and risk.

## 1. Introduction

Autoimmune thyroid diseases (AITD) comprise a group of organ-specific autoimmune disorders characterized by the presence of autoantibodies against thyroid antigens and lymphocyte infiltration.^[1]^ Clinically, AITD manifests as various forms of thyroid dysfunction, including hyperthyroidism and hypothyroidism.^[2]^ Hyperthyroidism, resulting from stimulating antibodies directed at the thyroid-stimulating hormone receptor, has a global prevalence ranging from 0.2% to 1.3%, with Graves’ disease (GD) being the most common etiology.^[3]^ In contrast, hypothyroidism, frequently associated with Hashimoto’s thyroiditis (HT), is characterized by the detection of specific autoantibodies such as thyroid peroxidase (TPOAb) and thyroglobulin (TgAb) antibodies, alongside lymphocytic infiltration in the thyroid.^[4]^

Although the underlying mechanisms remain to be fully elucidated, the pathogenesis of AITD involves an interplay of genetic and environmental factors.^[1,5]^ Immunoglobulin G (IgG), is a crucial component of humoral immunity, exhibits a well-characterized spectrum of N-glycan modifications. The N-glycans of IgG consist of a core structure formed by mannose and *N*-acetylglucosamine residues, with variable extensions including galactose, sialic acid, fucose, and bisecting *N*-acetylglucosamine (bisection).^[6–8]^ Altered IgG N-glycan profiles have been observed in various chronic inflammatory conditions, notably autoimmune diseases such as systemic lupus erythematosus,^[9,10]^ rheumatoid arthritis,^[11,12]^ inflammatory bowel disease,^[13]^ COVID-19,^[14]^ autoimmune pancreatitis,^[15,16]^ and multiple sclerosis.^[17]^ Nonetheless, the relationship between IgG N-glycosylation and AITD remains unclear, partly because observational studies and randomized controlled trials are often constrained by confounders and the possibility of reverse causality.

Inflammation, as a fundamental defense mechanism, involves the activation of immune cells and the production of cytokines, both of which are critical to the autoimmune response.^[18,19]^ Previous investigations have demonstrated strong correlations between inflammatory cytokines, immune cell phenotypes and the development as well as progression of AITD.^[20–24]^ Cytokine dysregulation can induce thyroid epithelial cell proliferation, abnormal differentiation, and immune dysfunction. Thereby contributing to the onset and progression of AITD.^[25]^ For example, patients with GD and HT have exhibited reduced level of CD8+ T cells, an increased CD4/CD8 ratio, and elevated populations of activated T cells expressing HLA-DR in peripheral blood. In thyroid tissue, both CD4+ and CD8+ cells infiltrate and remain activated, notably, CD4+ T cells have been implicated in mediating immune-related thyroid dysfunction.^[26]^ Circulating immune cells may, therefore, serve as key mediators in the pathogenesis of thyroid disorders.

Mendelian randomization (MR) is an analytical method that leverages genetic variants as instrumental variables (IVs) to infer causal relationships between risk factors and outcomes,^[27]^ thereby mitigating the impacts of confounding and reverse causation.^[28]^ In this study, publicly available genome-wide association study (GWAS) data were utilized to conduct an MR analysis, aiming to delineate potential causal associations between IgG N-glycosylation traits and 4 types of AITD. This approach further seek to elucidate the complex interrelationships among IgG N-glycosylation traits, inflammatory cytokines, immune cell phenotypes, and AITD.

## 2. Materials and methods

### 2.1. Study design

**Figure 1.**
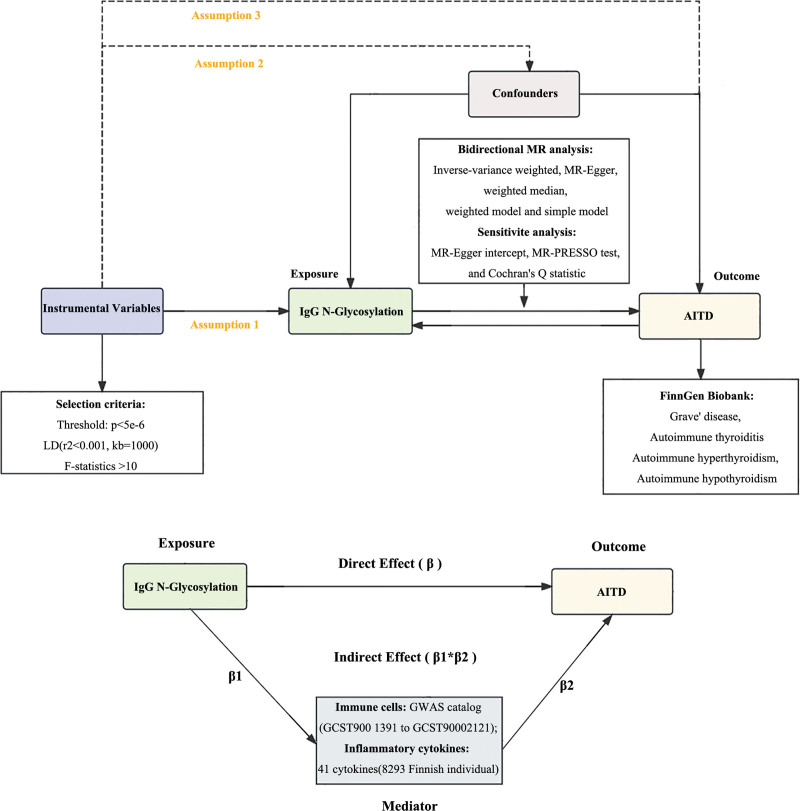
Overview of the Mendelian randomization (MR) analysis procedure. AITD = autoimmune thyroid disease, GWAS = genome-wide association study, IgG = immunoglobulin G, LD = linkage disequilibrium, MR = Mendelian randomization, MR-PRESSO = Mendelian randomization pleiotropy residual sum and outlier.

Figure 1 presents an overview of the MR analysis procedure. Initially, bidirectional 2-sample MR was employed to assess the causal relationships between the exposure and outcome. The MR approach relies on 3 fundamental assumptions: the genetic variants are robustly associated with the exposure; the genetic variants are no associated with potential any confounders that might distort the relationship between exposure and outcome; and the influence of the genetic variants on the outcome is mediated exclusively through the exposure. A reverse causality analysis was also performed to identify any reciprocal causal relationships. Subsequently, 2-sample MR was applied to investigate the mediation effects of potential mediators on the relationship between exposure and outcome by quantifying the effect sizes and proportions for each mediator. This study was conducted in accordance with the strengthening the reporting of observational studies in epidemiology using MR (STRBOE-MR) guidelines.^[29]^

### 2.2. Data source

The GWAS summary data for IgG N-glycosylation traits were obtained from a comprehensive meta-analysis of 4 cohorts of European ancestry: CROATIA-Korcula, CROATIA-Vis, ORCADES, and TwinsUK (N = 8090).^[30]^ The data, which are available at https://doi.org/10.7488/ds/2481 (accessed on April 22, 2024), comprise 77 ultra performance liquid chromatography (UPLC) IgG N-glycan traits (IGP1–77), including 23 directly measured N-glycosylation traits (IGP1–23) and 54 derived N-glycosylation traits (IGP24–77). Each directly measured peak primarily represents a major N-glycan structure, predominantly diantennary complex N-glycans, while the derived N-glycosylation traits described the abundance of specific glycan groups with common structural features (Table S1, Supplemental Digital Content, https://links.lww.com/MD/Q524).

The FinnGen study, a large-scale genomics collaboration, analyzed genetic variation in over 5,00,000 Finnish Biobank samples to investigate disease mechanisms and predispositions, which is a collaboration between research organizations and biobanks within Finland and international industry partners.^[31]^ We obtained summary data on GD, AT, autoimmune hypothyroidism (AH) and AH from FinnGen Biobank (https://www.finngen.fi/en). The GWAS dataset for GD, diagnosed with International Classification of Diseases-10 (ICD-10) code E05.0, included 3176 cases and 4,09,005 controls, including 2689 females and 487 males. For autoimmune hyperthyroidism (ICD-10 code E05.9), there were 1991 cases and 3,05,175 controls, consisting of 1647 females and 344 males. For AH (ICD-10 code E03.8), there were 45,320 cases and 2,98,847 controls, consisting of 36,229 female and 9091 male patients. Finally, the AT dataset, diagnosed with ICD-10 code E06.3, contained 539 cases and 3,49,717 controls, with 465 females and 74 males. Detailed data specifications are provided in Table 1.

**Table 1:** An overview of the summary data included in this study.

Cohort	Phenotypes	Sample size	Population ancestry	Data sources	ICD-10
Exposure	IgG N-glycosylation traits	8090	European	https://doi.org/10.7488/ds/2481	NA
Mediator	Immune cells	3757	European	GWAS Catalog	NA
Mediator	Inflammatory cytokines	8293	European	https://doi.org/10.1016/j.ajhg.2016.11.007	NA
Outcome	Graves’ disease	4,12,181	European	FinnGen Biobank (R10)	E05.0
Outcome	Autoimmune thyroiditis	3,50,256	European	FinnGen Biobank (R10)	E06.3
Outcome	Autoimmune hypothyroidism	3,44,168	European	FinnGen Biobank (R10)	E03.8
Outcome	Autoimmune hyperthyroidism	3,07,166	European	FinnGen Biobank (R10)	E05.9

GWAS = genome-wide association study, ICD-10 = International Classification of Diseases-10, IgG = immunoglobulin G.

This study also conducted mediation analysis using GWAS summary data on immune cell phenotypes and inflammatory cytokines. The immune cell phenotype data, covering 3757 individuals of European ancestry from nonoverlapping cohorts, encompassed 731 immune features, including absolute cell counts (n = 118), median fluorescence intensity values (n = 389), morphological parameters (n = 32), and relative cell counts (n = 192). These data were obtained from GWAS Catalog (entries GCST0001391 to GCST0002121)^[32]^ and include multiple cell types such as B cells, CDCs, T cells at different maturation stages, monocytes, bone marrow cells, TBNK (T cells, B cells, and Natural Killer cells), and Treg panels, with morphological parameters focusing on CDC and TBNK. Inflammatory cytokines GWAS data were obtained from the meta-analysis of summary statistics for 41 inflammatory cytokines, excluding BMI as a covariate – from the 3 cohorts (YFS, FINRISK 1997, and 2002), encompassing 8293 Finnish individuals.^[33]^ Further details regarding these datasets ares presented in Table 1.

### 2.3. Selection of genetic instrumental variables

For the selection of genetic IVs, single nucleotide polymorphisms (SNPs) that were strongly associated with exposure were chosen to assess causal relationships. A stringent genome-wide significance threshold (*P* < 5e−6) was applied to select IVs, ensuring that a sufficiently large number of SNPs were included. SNPs with linkage disequilibrium (*r*² < 0.001) within 10,000 kb were excluded to maintain independence among IVs. Furthermore, IVs were evaluated using the *F*-statistic, and those with an *F*-statistic < 10 were excluded due to insufficient genetic effects. Finally, the remaining IVs were selected for subsequent analyses.

### 2.4. Statistical analysis

Statistical analysis was conducted using bidirectional 2-sample MR employing 5 methods: the random-effects inverse-variance weighted (IVW), MR-Egger, weighted median, weighted mode and sample mode, using the “TwoSampleMR” package (version 0.6.15) in R software (version 4.3.1).^[27]^ The IVW method served as the primary statistical approach, with the remaining methods applied for supplementary analysis. The results were presented as odds ratios (ORs) with corresponding 95% confidence intervals (CIs). Associations were deemed statistically significant when the IVW *P*-value was < .05 and the direction of the IVW and MR-Egger analyses was consistent.

Heterogeneity among SNPs in the IVW method was assessed using Cochran’s *Q* test.^[34]^ Additionally, a leave-one-out analysis was performed to evaluate the impact of individual SNPs on the overall MR estimate.^[35]^ Further sensitivity analyses were conducted using scatter plots, and horizontal pleiotropy was detected using the MR-Egger intercept test and Mendelian randomization pleiotropy residual sum and outlier global test.^[36]^ A *P*-value > .05 indicated no horizontal pleiotropy, thereby supporting the validity of the IVW results.

We employed 2 mediation methods, 2-step MR and MVMR, to identify potential mediators of IgG N-glycosylation traits in the AITD. First, univariable MR was utilized to identify mediators causally influenced by exposure; their effect sizes (β1) were calculated accordingly. Next, univariable MR was reapplied to select mediators that exerted causal effects on the outcome, and their corresponding effect sizes (β2) were calculated. Mediators were retained based on logical consistency: if the total effect (β) was positive, both β1 and β2 were required to have the same sign; if β was negative, β1 and β2 were expected to have opposite signs. The mediated effects (β1 × β2) and corresponding proportions ((β1×β2)/β) were calculated using the “product of coefficients” method.^[37]^ Finally, MVMR was conducted using “Mendelian Randomization” package (version 0.10.0) as an alternative strategy to validate the mediating role of the metabolites identified via univariable MR. The IVW method served as the statistical approach, deeming associations significant when the IVW *P*-value was < .05. Horizontal pleiotropy was assessed with the MR-Egger intercept test, where a *P*-value > .05 indicated no pleiotropy. Additionally, heterogeneity analysis was performed using Cochrane *Q* statistics for both MR-egger and IVW, with a *P*-value > .05 indicating no significant heterogeneity.

## 3. Results

### 3.1. Identification and screening of instrumental variables

Following the selection of SNPs based on established criteria, the number of SNPs associated with IgG N-glycosylation ranged from 3 to 21 (median = 14). For immune cells, the number varied from 4 to -21 (median = 11), whereas for inflammatory cytokines, it spanned from 4 to 63 (median = 5). In the case of AITD, the number ranged from 9 to 266 (median = 35). Additionally, the F-statistics associated with these SNPs exhibited a wide range (from 10 to 42,272), underscoring the robustness of the selected IVs (Tables S2–S5, Supplemental Digital Content, https://links.lww.com/MD/Q524).

### 3.2. Causal associations of IgG N-glycosylation on AITD

**Figure 2.**
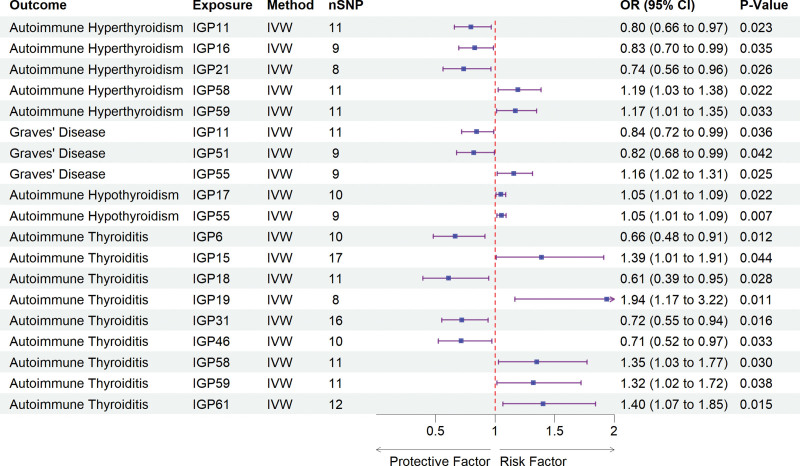
Forest plot illustrated the positive results from the MR analyses between IgG N-glycosylation traits and AITD. AITD = autoimmune thyroid disease, CI = confidence interval, IgG = immunoglobulin G, IVW = inverse-variance-weighted, MR = Mendelian randomization, OR = odds ratios, SNP = single nucleotide polymorphisms.

Among the 77 IgG N-glycan traits analyzed, 15 (IGP6, IGP11, IGP15, IGP16, IGP17, IGP18, IGP19, IGP21, IGP31, IGP46, IGP51, IGP55, IGP58, IGP59, and IGP61) exhibited causal relationships with AITD, according to the IVW method. Figure 2 illustrates the causal effects of these traits on the 4 types of AITD.

For autoimmune hyperthyroidism, significant positive associations were observed for IGP58 (OR: 1.19, 95% CI: 1.03–1.38; *P* = .022) and IGP59 (OR: 1.17, 95% CI: 1.01–1.35; *P* = .033), whereas, IGP11 (OR: 0.80, 95% CI: 0.66–0.97; *P* = .023), IGP16 (OR: 0.83, 95% CI: 0.70–0.99; *P* = .035), and IGP21 (OR: 0.74, 95% CI: 0.56–0.96; *P* = .026) exhibited protective effects. When AH was considered as the outcome, only IGP17 (OR: 1.05, 95% CI: 1.01–1.09; *P* = .022) and IGP55 (OR: 1.05, 95% CI: 1.01–1.09; *P* = .007) exhibited were associated with an increased risk. In the case of GD, IGP55 was associated with elevated risk (OR: 1.15, 95% CI: 1.03–1.38; *P* = .025), while IGP11 (OR: 0.84, 95% CI: 0.72–0.99; *P* = .023) and IGP51 (OR: 0.82, 95% CI: 0.68–0.99; *P* = .042) demonstrated inverse associations. For considering AT, 5 glycan traits – IGP15 (OR: 1.39, 95% CI: 1.01–1.91; *P* = .044), IGP19 (OR: 1.94, 95% CI: 1.17–3.22; *P* = .011), IGP58 (OR: 1.35, 95% CI: 1.03–1.77; *P* = .030), IGP59 (OR: 1.32, 95% CI: 1.02–1.72; *P* = .038), and IGP61 (OR: 1.40, 95% CI: 1.07–1.85; *P* = .015) – were associated with an increased risk, whereas IGP6 (OR: 0.66, 95% CI: 0.48–0.91; *P* = .012), IGP18 (OR: 0.61, 95% CI: 0.39–0.95; *P* = .028), IGP31 (OR: 0.72, 95% CI: 0.55–0.94; *P* = .016), and IGP46 (OR: 0.71, 95% CI: 0.52–0.97; *P* = .033) exhibited protective effects (Table S6, Supplemental Digital Content, https://links.lww.com/MD/Q524). Moreover, the Cochrane *Q* test indicated no significant heterogeneity among the IVs, and both the MR-Egger intercept test and Mendelian randomization pleiotropy residual sum and outlier provided no evidence of horizontal pleiotropy, thereby affirming the robustness of the MR findings (Table S7, Supplemental Digital Content, https://links.lww.com/MD/Q524).

### 3.3. Causal associations of AITD on IgG N-glycosylation

**Figure 3.**
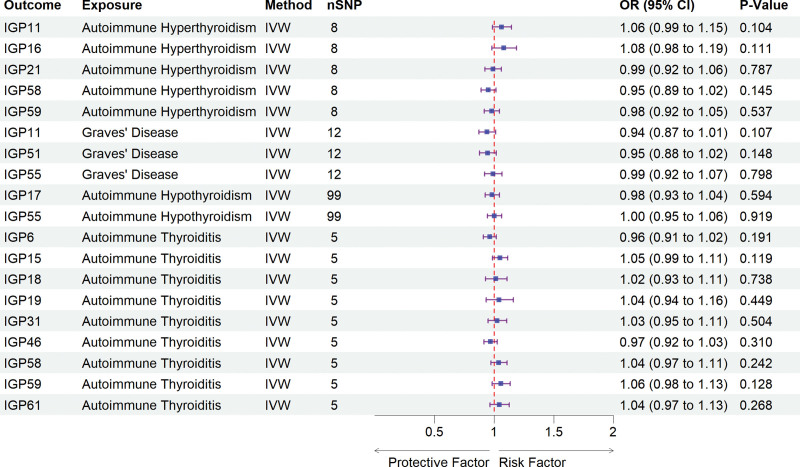
Forest plot illustrated the positive results from the reverse MR analyses between IgG N-glycosylation traits and AITD. AITD = autoimmune thyroid disease, CI = confidence interval, IgG = immunoglobulin G, IVW = inverse-variance-weighted, MR = Mendelian randomization, OR = odds ratios, SNP = single nucleotide polymorphisms.

To assess potential reverse causation between IgG N-glycosylation traits and AITD, we conducted a reverse MR analysis. This analysis, primarily utilized the IVW method, treating the previously identified IgG N-glycosylation traits as outcomes and AITD as exposures. Our findings demonstrated no significant causal relationship between IgG N-glycosylation traits and AITD, ruling out reverse causality and supporting a robust foundation for subsequent mediation analyses (Fig. 3, Tables S8 and S9, Supplemental Digital Content, https://links.lww.com/MD/Q524).

### 3.4. Mediation analyses of potential mediators

**Figure 4.**
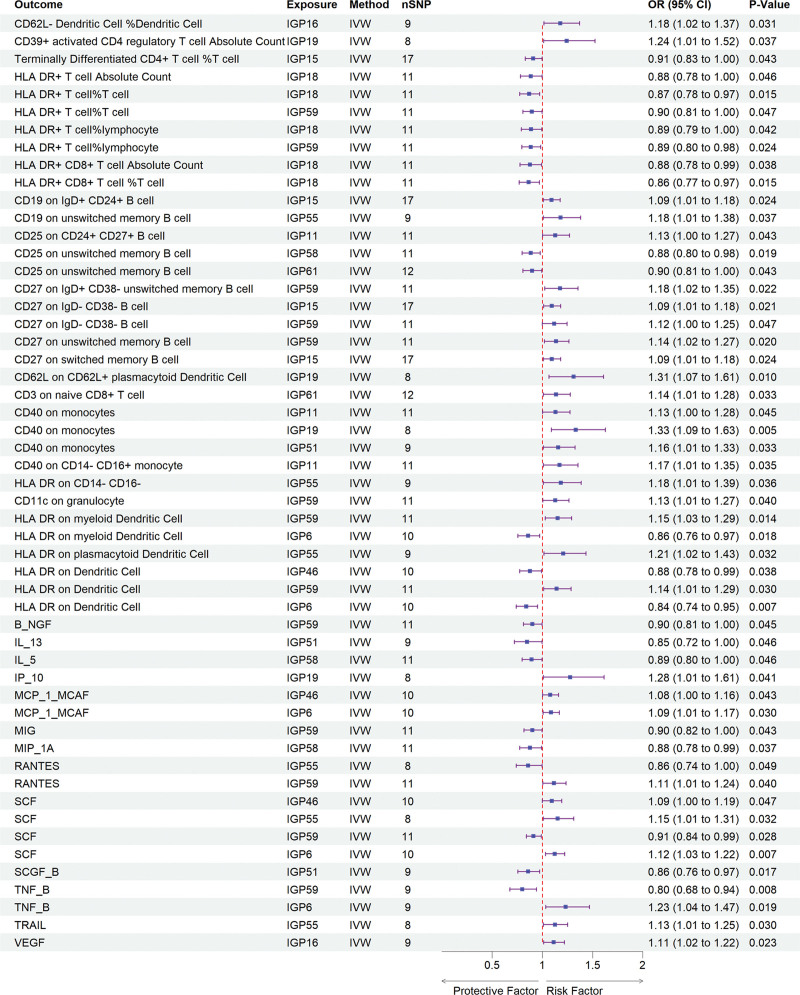
Forest plot illustrated the positive results from the MR analyses between the IgG N-glycosylation trait and mediators (immune cells and inflammatory cytokines). CI = confidence interval, IgG = immunoglobulin G, IVW = inverse-variance-weighted, MR = Mendelian randomization, OR = odds ratios, SNP = single nucleotide polymorphisms.

**Figure 5.**
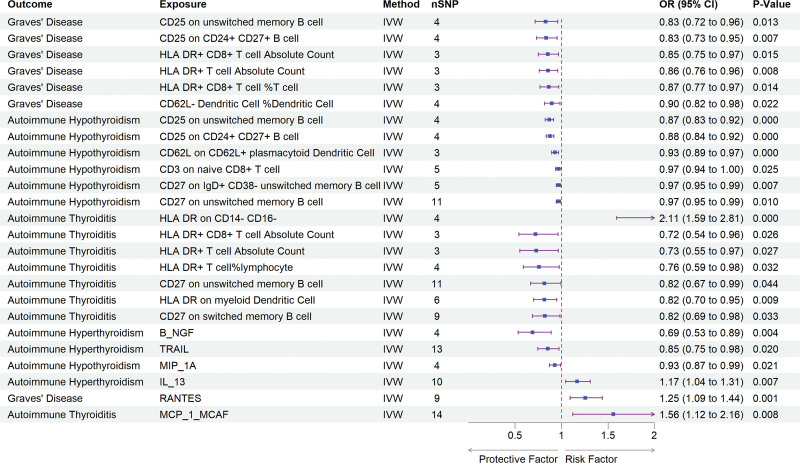
Forest plot illustrated the positive results from the MR analyses between the mediators (immune cells and inflammatory cytokines) and AITD. AITD = autoimmune thyroid disease, CI = confidence interval, IgG = immunoglobulin G, IVW = inverse-variance-weighted, MR = Mendelian randomization, OR = odds ratios, SNP = single nucleotide polymorphisms.

**Figure 6.**
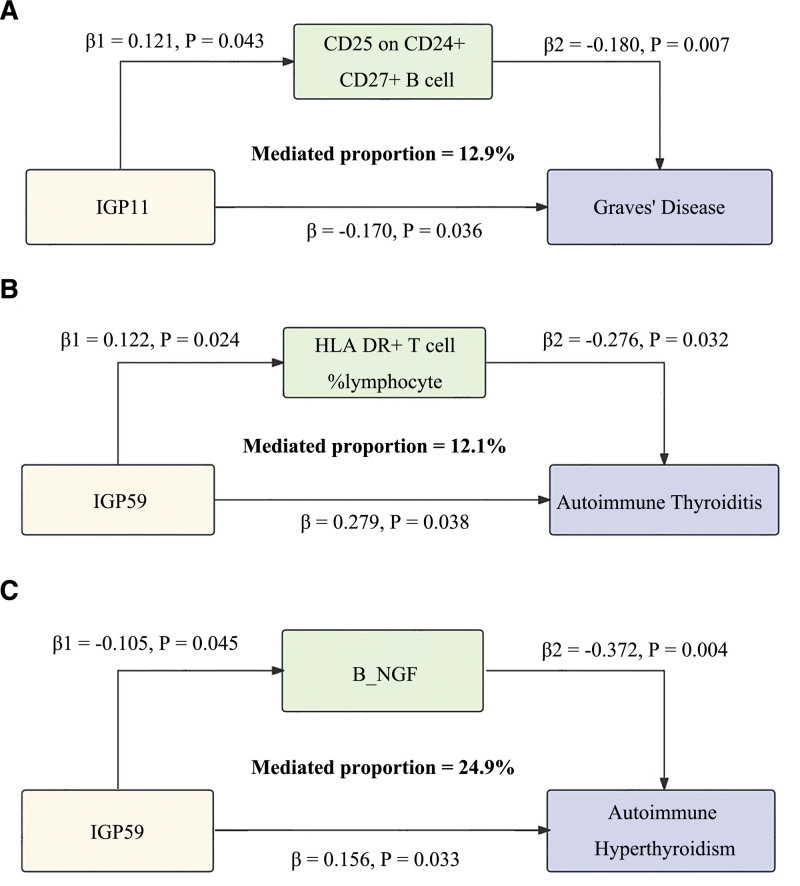
Mediation effects and proportion mediated by immune cells (A, B) and inflammatory cytokine (C).

To investigate the possible mediating effects of inflammatory cytokines and immune cell phenotypes on the causal association between IgG N-glycosylation traits and AITD, we conducted a 2-step mediation analysis. In the first step, univariable MR analyses were conducted using 15 IgG N-glycosylation traits (derived from previous MR analyses) as exposures and 41 inflammatory cytokines along with 731 immune cell phenotypes as outcomes to estimate effect sizes (β1). Among these, 13 inflammatory cytokines and 25 immune cell phenotypes exhibited significant causal relationships with the IgG N-glycosylation traits (Fig. 4, Tables S10–S13, Supplemental Digital Content, https://links.lww.com/MD/Q524). In the subsequent step, we excluded significant genetic IVs to evaluate the impact of 6 inflammatory cytokines and 14 immune cell phenotypes on AITD (β2). Our analysis revealed that “HLA DR on CD14- CD16-” and IL-13, RANTES, and MCP_1_MCAF significantly increased the risk of AITD, whereas other immune cell phenotypes and B_NGF, TRAIL, and MIP_1A significantly decreased the risk (Fig. 5, Tables S14–S17, Supplemental Digital Content, https://links.lww.com/MD/Q524). Finally, we identified key mediators, including “CD25 on CD24+ CD27+ B cell” (mediation proportion: 12.9%), “HLA DR+ T cell%lymphocyte” (mediation proportion: 12.1%), and B_NGF (mediation proportion: 24.9%), which mediate the causal associations between IGP11 and GD, IGP59 and AT, and IGP59 and autoimmune hyperthyroidism, respectively (Fig. 6). In the MVMR analysis, after adjusting for the influence of B_NGF, IGP59 remained significantly associated with autoimmune hyperthyroidism. MR-Egger analysis indicated no evidence of horizontal pleiotropy (intercept *P* = .887), and Cochrane’s *Q* test demonstrated no heterogeneity (*P* = .517), supporting the robustness of these findings. The other 2 mediators derived from the univarible MR analysis did not yield significant results in the MVMR analysis, with either an IVW *P*-value > .05 or a pleiotropy test showing a *P*-value < .05. Detailed information is presented in Tables 2 and S18, Supplemental Digital Content, https://links.lww.com/MD/Q524.

**Table 2:** Mediation analysis of the effect of IgG N-glycosylation on AITD via immune cells and inflammatory cytokines using 2-step MR and MVMR.

Exporsure	Mediator	Outcome	Total effect (β)	Effect of exposure on mediator (β1)	Effect of mediator on outcome (β2)	Indirect effect (β1*β2)	Proportion mediated (β1*β2/β), %	MVMR-IVW	MR-Egger		Heterogeneity	
								*P*	Intercept	*P*	Cochrane *Q*	*P*
IGP11	CD25 on CD24+ CD27+ B cell	Graves’ disease	−0.170	0.121	−0.180	−0.022	12.89	.080	0.017	.194	25.67	.042
IGP59	HLA DR+ T cell%lymphocyte	Hashimoto’s thyroiditis	0.279	−0.122	−0.276	0.034	12.07	.024	0.117	.027	2.01	.991
IGP59	B_NGF	Autoimmune hyperthyroidism	0.156	−0.105	−0.372	0.039	24.87	.013	0.004	.887	8.17	.517

AITD = autoimmune thyroid disease, IgG = immunoglobulin G, IVW = inverse-variance-weighted, MR = Mendelian randomization, MVMR = multivariable Mendelian randomization.

## 4. Discussion

This large-scale GWAS investigated the role of IgG N-glycosylation in the etiology and pathogenesis of AITD. Builds upon previous findings that alterations in IgG N-glycosylation patterns are associated with varying degrees of thyroid inflammation severity and disease progression.^[38–41]^ The study identified IGP55, the percentage of agalactosylated structures in total neutral IgG glycans, as a significant predictor of GD. This observation corroborates earlier evidence that elevated levels of agalactosylated structures with proximal fucose and oligomannose are linked to an increased risk of GD.^[40]^ Moreover, the study highlights the role of core fucosylation in modulating IgG N-glycosylation patterns, noting a reduction in core fucosylation levels among AITD patients, including HT, compared with healthy controls.^[39,40]^ Additional analyses revealed that IGP15, IGP59 and IGP61 are significantly associated with an increased risk of AT, suggesting that fucosylated, galactosylated, monosialylated and digalactosylated IgG structures may influence the anti-inflammatory activity and pathogenesis of HT, consistent with the findings of Ząbczyńska et al.^[41]^ Conversely, while Martin et al reported reduced core fucosylation in IgG glycans in patients with AITD, including HT.^[39]^ Trzos et al demonstrated decreases in 2 specific core-fucosylated IgG structures alongside an increase in the F(6)A2 N-glycan in HT patients.^[40]^ These contrasting finding suggest that although certain glycosylation features may be diminished, others are enhanced, potentially reflecting compensatory mechanisms or distinct pathogenic pathways. Collectively, these results underscore the potential of IgG glycosylation as a biomarker for AITD and implicate specific glycosylation patterns in the disease’s development and progression.

The study also identified 13 inflammatory cytokines and 25 immune cell phenotypes that exhibiting a significant causal relationship with IgG N-glycosylation traits. Further analyses established significant associated between AITD and 6 inflammatory cytokines, including B_NGF, IL_13, MCP_1_MCAF, MIP_1A, RANTES, TRAIL, as well as 14 immune cell phenotypes, including HLA-DR+ CD8+T cells, HLA DR+ T cells, myeloid dendritic cells, plasmacytoid dendritic cells, memory B cells, and other immune cell phenotypes. A recent MR study revealed that elevated circulating levels of TNF-β, IL-12p70, IL-13, and IFN-γ, along with decreased levels of SCGF-β, MCP-1 and TNF-α are associated with a higher risk of GD/H.^[42]^ Notably, IL_13 and MCP_1, which are central to immune responses and inflammatory processes,^[43–46]^ may regulate differentiation, growth, and secretory functions of thyroid cells via endocrine, autocrine, and paracrine mechanisms. In addition, immune cell phenotypes related to major histocompatibility complex class II molecules, such as HLA-DR expression of on HLA-DR+ CD4+ T cells and HLA-DR+ T cells, have been linked to a decreased risk of hypothyroidism.^[47]^ Complementary studies have reported a reduction in peripheral blood CD8+ T cells in AITD patients and an increased risk of HT associated with a higher proportion of CD62L−plasmacytoid DC, with additional evidence linking increased plasmacytoid dendritic cell density to disease severity.^[48]^ These findings reinforce the importance of immune cell phenotypes in the pathogenesis of AITD and highlight the potential utility of immune cell biomarkers in clinical practice.

Mediation analysis demonstrated that IGP59 may promote AT through the inflammatory cytokine β-nerve growth factor (β-NGF), suggesting that either IGP59 or β-NGF could serve as potential biomarkers for the early diagnosis and clinical management of AT. β-NGF, a neurotrophic factor, not only stimulates the growth, differentiation, and maturation of neurons but also directly influences immune responses, modulating the activity of B and T cells.^[49]^ Elevated levels of β-NGF have been observed in individuals with AITD, and β-NGF can activate mast cells, thereby exacerbating the inflammatory state and increasing the risk of AITD.^[50]^ Yao et al demonstrated that GD can elevate blood levels of β-NGF.^[42]^

Our study further demonstrated that IGP11 and IGP59 exert inhibitory and promotional effects, respectively, on GD and AT through specific immune cell phenotypes, namely, “CD25+ on CD24+ CD27+ B cells” and “HLA DR+ T cell % lymphocytes” as determined by univarible MR analysis. These results suggest that high CD25 expression in B cells may be associated with a lower risk of GD, even though MVMR analysis did not yield significant results. Although previous limited studies have suggested that elevated CD25 levels are linked to poorer clinical prognoses in patients with B cell malignancies,^[51]^ further investigations are warranted to elucidate the relationship between these immune phenotypes and AITD. In addition, CD4+ T cells play a regulatory role in the immune response to AITD, and HLA-DR expression – commonly associated with T cell activation – indicates its involvement in immune regulation and disease control in AH.^[47]^ Our findings indicate that HLA-DR+ T cells are associated with a reduced risk of AT, which aligns with the observations reported by Cao et al.^[47]^ Nonetheless, further research is required to fully understand the underlying mechanisms and to identify potential therapeutic targets.

This study integrated large-scale GWAS data pertaining to IgG N-glycosylation, inflammatory cytokines, immune cells, and AITD, employing a rigorous analytical framework to investigate the causal relationships between IgG N-glycosylation and 4 types of AITD. However, several limitations should be noted. First, although the study identified potential mediators in the causal relationship between IgG N-glycosylation and AITD, the inherent complexity of the biological pathways likely resulted in incomplete coverage of all possible mediation routes. Second, the restriction of the study population to individuals of European ancestry may limit the generalizability of the findings. Third, the intentional omission of multiple testing corrections, such as Bonferroni or false discovery rate adjustments, was aimed at prioritizing the identification of potential biomarkers or therapeutic targets for AITD, with the recognition that strict Bonferroni criteria might overlook biologically significant indicators. Final, the control group in the FinnGen dataset may include individuals with autoimmune conditions unrelated to thyroid disease, potentially introducing collider bias. However, our data do not allow us to rule out this possibility, nor do they enable the performance of appropriate sensitivity analyses to address it. Future research should address these limitations by expanding the study populations and incorporating appropriate statistical corrections to enhance our understanding of the complex interplay involved, ultimately informing guiding the development of novel diagnostic and therapeutic strategies for AITD.

## 5. Conclusions

Our study provides valuable insights into the potential causal role of genetically predicted IgG N-glycans in AITD. By comprehensively evaluating the associations among IgG glycosylation, inflammatory cytokines, immune cell phenotypes, and AITD, we identified key biomarkers for predictive of AITD risk and prognosis. Further investigations are warranted to expand our understanding and to develop novel diagnostic and therapeutic strategies for these autoimmune conditions.

## Acknowledgments

We would like to express our sincere gratitude to the providers of the public data utilized in this study, including the FinnGen Biobank for genome-wide association study (GWAS) data and the IEU Open GWAS project for supplying essential data on IgG N-glycosylation, autoimmune thyroid diseases, immune cells, and inflammatory cytokines. We also thank the researchers and the participants involved in this study.

## Author contributions

**Conceptualization:** Wenhua Li, Ying Zhang.

**Data curation:** Wenhua Li, Ying Zhang, Min Yi.

**Formal analysis:** Wenhua Li, Dongni Fan, Dongmi Wei, Wei Wei.

**Funding acquisition:** Min Yi.

**Investigation:** Wenhua Li, Dongni Fan, Dongmi Wei, Wei Wei.

**Methodology:** Ying Zhang.

**Project administration:** Wenhua Li.

**Software:** Wenhua Li.

**Supervision:** Wenhua Li, Ying Zhang, Min Yi.

**Validation:** Wenhua Li, Ying Zhang.

**Visualization:** Wenhua Li, Ying Zhang.

**Writing – original draft:** Wenhua Li, Dongni Fan, Dongmi Wei, Wei Wei.

**Writing – review & editing:** Wenhua Li, Ying Zhang, Min Yi.

## Supplementary Material


